# Natural geochemical markers reveal environmental history and population connectivity of common cuttlefish in the Atlantic Ocean and Mediterranean Sea

**DOI:** 10.1098/rsif.2020.0309

**Published:** 2020-07-29

**Authors:** Jay R. Rooker, R. J. David Wells, Piero Addis, Haritz Arrizabalaga, Miguel Baptista, Giovanni Bearzi, Michael A. Dance, Igaratza Fraile, Thomas Lacoue-Labarthe, Jessica M. Lee, Persefoni Megalofonou, Rui Rosa, Ignacio Sobrino, António V. Sykes, Roger Villanueva

**Affiliations:** 1 Department of Marine Biology, Texas A&M University, 1001 Texas Clipper Road, Galveston, TX 77553, USA; 2 Department of Environmental and Life Science, University of Cagliari, Via Fiorelli 1, 09126 Cagliari, Italy; 3 AZTI, Marine Research, Basque Research and Technology Alliance (BRTA), Herrera Kaia, Portualdea z/g, 20110 Pasaia – Gipuzkoa, Spain; 4 MARE - Marine and Environmental Sciences Centre, Laboratório Marítimo da Guia, Faculdade de Ciências, Universidade de Lisboa, Lisboa, Avenida Nossa Senhora do Cabo 939, 2750-374 Cascais, Portugal; 5 Dolphin Biology and Conservation, Cordenons, Pordenone, Italy; 6 Department of Oceanography and Coastal Sciences, Louisiana State University, 2255 Energy, Coast and Environment Building, Baton Rouge, LA 70803, USA; 7 Littoral Environnement et Sociétés (LIENSs) - UMR 7266 Bâtiment ILE, 2, rue Olympe de Gouges, 17000 La Rochelle, France; 8 Department of Biology, National and Kapodistrian University of Athens,15784 Athens, Greece; 9 Instituto Español de Oceanografía, Puerto Pesquero s/n, 11006, Cádiz, Spain; 10 Center of Marine Sciences, Universidade do Algarve Campus de Gambelas, 8005-139 Faro, Portugal; 11 Institut de Ciències del Mar (CSIC), Passeig Maritim 37-49, 08003 Barcelona, Spain

**Keywords:** cephalopod, migration, cuttlefish, geochemistry, population structure

## Abstract

Natural markers (δ^13^C and δ^18^O stable isotopes) in the cuttlebones of the European common cuttlefish (*Sepia officinalis*) were determined for individuals collected across a substantial portion of their range in the Northeast Atlantic Ocean (NEAO) and Mediterranean Sea. Cuttlebone δ^13^C and δ^18^O were quantified for core and edge material to characterize geochemical signatures associated with early (juvenile) and recent (sub-adult/adult) life-history periods, respectively. Regional shifts in cuttlebone δ^13^C and δ^18^O values were detected across the 12 sites investigated. Individuals collected from sites in the NEAO displayed more enriched δ^13^C and δ^18^O values relative to sites in the Mediterranean Sea, with the latter also showing salient differences in both markers among western, central and eastern collection areas. Classification success based on cuttlebone δ^13^C and δ^18^O values to four geographical regions (NEAO, western, central and eastern Mediterranean Sea) was relatively high, suggesting that environmental conditions in each region were distinct and produced area-specific geochemical signatures on the cuttlebones of *S. officinalis*. A modified δ^13^C and δ^18^O baseline was developed from sites proximal to the Strait of Gibraltar in both the NEAO and Mediterranean Sea to assess potential mixing through this corridor. Nearly, all (95%) of δ^13^C and δ^18^O signatures of *S. officinalis* collected in the area of the NEAO closest to the Strait of Gibraltar (Gulf of Cadiz) matched the signatures of specimens collected in the western Mediterranean, signifying potential movement and mixing of individuals through this passageway. This study extends the current application of these geochemical markers for assessing the natal origin and population connectivity of this species and potentially other taxa that inhabit this geographical area.

## 1. Background

The European common cuttlefish (*Sepia officinalis*) is an important fishery resource throughout the Mediterranean Sea and adjacent waters of the Northeast Atlantic Ocean (NEAO) [1,2]. Due to large temporal shifts in the abundance and recruitment of *S. officinalis*, particularly for high-yield fisheries in certain areas (e.g. English Channel, Bay of Biscay [1]), there is renewed interest in their movement ecology and population connectivity [3]. This is primarily due to the fact that spatio-temporal shifts in distribution of marine organisms, particularly spawning stock biomass (SSB), can have profound implications for the management of harvested fisheries [4]. Current knowledge on the movement of *S. officinalis* is limited, preventing the implementation of conservation measures (e.g. time–area closures) for protecting highly suitable habitat or key areas used by spawning adults. Several different approaches have been used to clarify the movements and home range of *S. officinalis,* including conventional tagging [5], acoustic telemetry [6] and spatial shifts in catch from fishery-dependent data [7]. More recently, Dance *et al*. [8] used natural tracers in cuttlebones to investigate small-scale habitat shifts of this species in the Adriatic Sea. While earlier studies have shed important light on the habitat use and movement of *S. officinalis*, expanding the geographical scope of our knowledge on population connectivity is critical to their management.

Cuttlefish (class Cephalopoda) are characterized by the presence of an aragonitic internal shell (cuttlebone), and this gas-filled structure serves as a buoyancy control mechanism [9–11]. Similar to other biogenic carbonates found in cephalopods (statoliths) and fishes (otoliths), these aragonitic structures accrete material in a predictable manner and the microstructure retains information that records the timing of deposition [12]. In addition, the chemistry of material accreted in cuttlebones and other biogenic carbonates reflects ambient elemental or isotopic concentrations in seawater, and thus serve as natural markers for retrospective determination of past occurrences or environmental histories [8,13]. As a result, chemical markers in cuttlebones represent a powerful tool for predicting the natal origin, movement and population connectivity of cuttlefish [14].

The aim of the present study was to characterize regional variation in the geochemistry of cuttlebones of *S. officinalis* across a large section of its range in the NEAO and Mediterranean Sea. A common class of natural markers (stable C and O isotope ratios) was employed to determine whether distinct geochemical gradients exist within the Mediterranean Sea and areas outside this basin in the NEAO. We quantified δ^13^C and δ^18^O values for core and edge portions of the cuttlebone to contrast signatures and regional discrimination of habitats used during early (natal) and recent (time of collection) life-history periods, respectively. Multiple locations were sampled within each of four major geographical regions investigated (NEAO and western, central, and eastern Mediterranean Sea), and regional discrimination (classification success) was determined for both cuttlebone core and edge signatures. We then used cuttlebone core (δ^13^C_CORE_ and δ^18^O_CORE_) values to predict the natal origin of sub-adult/adult *S. officinalis* collected from a potential mixing area at the juncture between the NEAO and Mediterranean Sea. The spatial configuration of distinct geochemical provinces within areas investigated provides important insights into the suitability of these natural markers for addressing related ecological questions (ontogenetic habitat shifts) as well as population connectivity within the NEAO and Mediterranean Sea.

## 2. Methods

**Figure 1.**
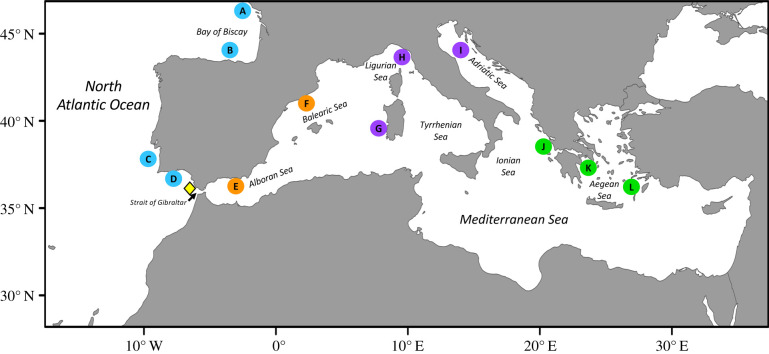
Map of collection sites (*n* = 12) for *Sepia officinalis* in the Northeast Atlantic Ocean and Mediterranean Sea. Sites grouped into four major geographical areas: Northeast Atlantic Ocean, NEAO (Bay of Biscay to Gulf of Cadiz), western Mediterranean Sea, W-MED (Alboran Sea to Balearic Sea), central Mediterranean Sea, C-MED (Ligurian Sea to Adriatic Sea), eastern Mediterranean Sea, E-MED (Ionian Sea to Aegean Sea). Yellow symbol (diamond) indicates the collection region in close proximity to the Strait of Gibraltar (Gulf of Cadiz) that was used to assess potential movement of individuals between the NEAO and W-MED.

**Figure 2.**
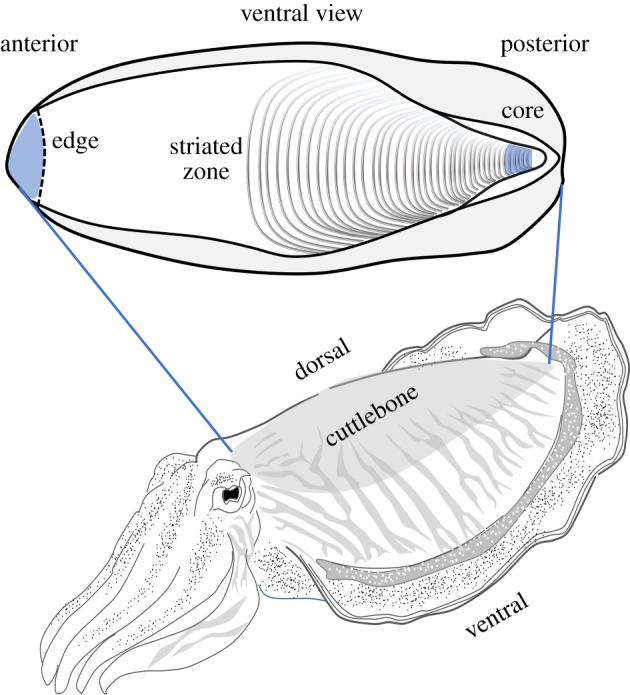
Diagram showing the position of the cuttlebone on *Sepia officinalis* and the ventral view of the cuttlebone with the approximately 1 cm sampling location used to determine δ^13^C and δ^18^O values for core and edge material denoted with blue shading.

*Sepia officinalis* were collected between February 2013 and February 2014 across several locations in the NEAO and Mediterranean Sea, with samples falling into four major geographical regions: NEAO [Bay of Biscay to Gulf of Cadiz], western Mediterranean Sea, W-MED [Alboran Sea to Balearic Sea], central Mediterranean, C-MED [Ligurian Sea to Adriatic Sea], eastern Mediterranean, E-MED [Ionian Sea to Aegean Sea] (figure 1). Specimens were almost exclusively sub-adult/adult *S. officinalis* (table 1), and obtained from commercial trawlers, government run sampling programmes, artisanal fishers, and local fish markets. Cuttlebones were extracted, cleaned and dried in the laboratory. Core and edge material for δ^13^C and δ^18^O analysis was collected using the protocol described previously [8]. Briefly, approximately 1-cm sections (approx. 1–3+ month interval [15]) of material were isolated from the posterior origin (core) and anterior margin (edge) of each cuttlebone (figure 2). For each section, material was carefully removed from the phragmocone portion of the cuttlebone by sampling from the ventral surface upward to the hypostracum (ventral layer of the dorsal shield). Material was isolated from each cuttlebone with a scalpel and powdered.

**Table 1.** Summary of *Sepia officinalis* collected in the Northeast Atlantic Ocean and Mediterranean Sea with information on site code (A-L), location, collection date, sample number (N) and mean mantle length (ML) of specimens from each site. ♢ Collection area in Bay of Cadiz outside Strait of Gibraltar used to assess exchange between the Northeast Atlantic Ocean and Mediterranean Sea.

	location or	collection date		mean ML
site^a^	sampling port	(MO/YR)	N	±1 s.d. (mm)
A	La Rochelle, France	June 2013	20	143.7 (±9.4)
B	San Sebastian, Spain	February–June 2013	44	121.0 (±11.3)
C	Cascais, Portugal	June 2013	24	79.2 (±36.9)
D	Olhão, Portugal	June 2013	19	79.8 (±16.3)
♢	Cadiz, Spain	June 2013	20	110.0 (±18.9)
E	Marbella, Spain	June 2013	10	111.4 (±20.3)
F	Cubellas, Spain	June 2013	19	91.8 (±7.8)
G	Sardinia, Italy	September–October 2013	20	69.0 (±21.7)
H	La Spezia, Italy	October 2013	22	60.5 (±9.0)
I	Venice, Italy	October 2013	17	52.1 (±4.9)
J	Galaxidi, Greece	July–September 2013	20	103.0 (±16.9)
K	Athens, Greece	February 2014	20	88.3 (±16.5)
L	Santorini, Greece	February 2014	6	98.3 (±12.4)

^a^Denoted on figure 1.

Cuttlebone δ^13^C and δ^18^O were determined using an automated carbonate preparation device (KIEL-III Thermo Fisher Scientific, Inc.) coupled to an isotope ratio monitoring mass spectrometer (Finnigan MAT 252 Thermo Fisher Scientific, Inc.) at the University of Arizona Environmental Isotope Laboratory. Powdered cuttlebone samples were reacted with dehydrated phosphoric acid under vacuum at 70°C. The isotope ratio measurement was calibrated based on repeated measurements of the National Bureau of Standards (NBS), NBS-19 and NBS-18, with six standards run for every 40 samples; precision was approximately 0.08‰ and 0.11‰ for δ^13^C and δ^18^O, respectively. Cuttlebone δ^13^C and δ^18^O are reported relative to the Vienna Pee Dee Belemnite (VPDB) scale after comparison to an in-house laboratory standard calibrated to VPDB.

Multivariate analysis of variance (MANOVA) was used to test for regional difference in cuttlebone core and edge δ^13^C and δ^18^O. In addition, univariate contrasts (ANOVAs) were performed on individual markers to assist in the identification of the most influential markers for regional discriminations. MANOVA significance used Pillai's trace statistic, and significance for all ANOVA and MANOVA tests was based on an alpha level of 0.05. Results from ANOVA tests were examined further with Tukey's honestly significant difference (HSD) test to determine whether group means between specific regions were different. Paired *t*-tests were also performed between core and edge values for both δ^13^C and δ^18^O to determine whether early (core) and recent (edge) life-history signatures from the same individual differed. Canonical discriminant analysis (CDA) was used to display multivariate means of cuttlebone δ^13^C and δ^18^O, and discriminant function coefficients were incorporated into CDA plots as vectors from a grand mean to show the discriminatory influence of δ^13^C and δ^18^O by region. Quadratic discriminant function analysis (QDFA) was then used to determine the classification accuracy of cuttlebone δ^13^C and δ^18^O signatures for assigning individuals to their respective region (i.e. collection area). We selected QDFA over other multivariate approaches to assess classification success based on natural makers because it is preferred when the variance–covariance matrix of our predictor variables differs. Also, QDFA does not have the assumption of homogeneity of covariance matrices and is robust to moderate deviations from normality [16].

Natal origin of *S. officinalis* collected near the Strait of Gibraltar in the NEAO was predicted using direct maximum-likelihood estimates (MLE) of stock composition [17,18]. Mixed-stock analysis was run under bootstrap mode to obtain standard deviations around estimated proportions (error terms) with 500 simulations [19]. For our assessment of NEAO and W-MED mixing, we limited our source populations to the two nearest collection sites on each side of the Strait of Gibraltar (NEAO = sites C, D; W-MED = sites E, F; figure 1), and our unknown population was based on larger (greater than 100 mm mantle length) *S. officinalis* collected proximal to the Strait of Gibraltar in the Bay of Cadiz (distance approx. 80 km). Cuttlebone δ^13^C and δ^18^O values of *S. officinalis* collected in the Bay of Cadiz were all within 95% confidence ellipses of individuals from the two general regions used to create the baseline and thus we assumed that bias due to the presence of individuals from nurseries not included or not sampled was minimal.

## 3. Results

### 3.1. Regional variation in cuttlebone δ^13^C and δ^18^O

**Figure 3.**
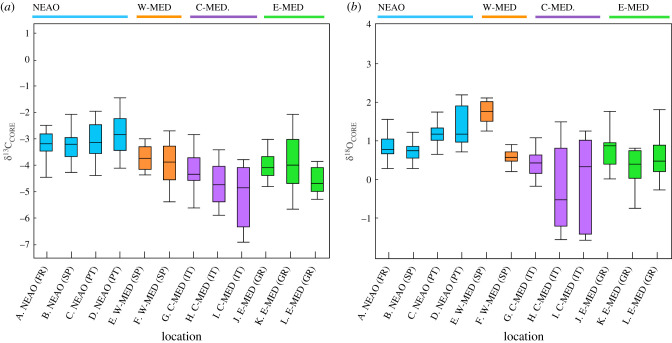
Box plot of cuttlebone (*a*) δ^13^C_CORE_ and (*b*) δ^18^O_CORE_ values by site and region for *Sepia officinalis*. Different colour box plots shown for the four major regions.

Significant spatial differences in cuttlebone δ^13^C_CORE_ and δ^18^O_CORE_ signatures (early life history) were detected for *S. officinalis* among the collection sites (MANOVA, *p* < 0.01; figure 3). Mean cuttlebone δ^13^C_CORE_ values ranged from −2.69‰ to −5.10‰ across the 12 sites, and a distinct trend was observed for *S. officinalis* from sampling sites in the NEAO versus sites within the Mediterranean Sea. Individuals collected in the NEAO generally displayed more enriched cuttlebone δ^13^C_CORE_ values (mean ± 1 SD; −3.16‰ ± 0.67‰) relative to all three regions in the Mediterranean Sea: W-MED (−3.94‰ ± 0.78‰), C-MED (−4.65‰ ± 0.95‰) and E-MED (−3.97‰ ± 0.78‰). Univariate contrasts indicated that cuttlebone δ^13^C_CORE_ values were significantly different among the four regions (ANOVA, *p* < 0.01), and Tukey HSD test indicated that δ^13^C_CORE_ values for *S. officinalis* from the NEAO region were significantly different from all three regions within the Mediterranean Sea. Site-specific variation in cuttlebone δ^18^O_CORE_ was also present and mean values ranged from −0.2‰ to 1.3‰ across the 12 sites sampled. Similar to δ^13^C_CORE_, cuttlebone δ^18^O_CORE_ values were significantly different among the four geographical regions (ANOVA, *p* < 0.01), and mean values were higher in the NEAO (0.92‰ ± 0.44‰) compared to sites within the Mediterranean Sea: W-MED (0.53‰ ± 0.37‰), C-MED (0.04‰ ± 0.95‰) and E-MED (0.62‰ ± 0.67‰) Again, Tukey HSD tests indicated that δ^18^O_CORE_ values for individuals collected in the NEAO region were significantly different from all three regions of the Mediterranean Sea.

**Figure 4.**
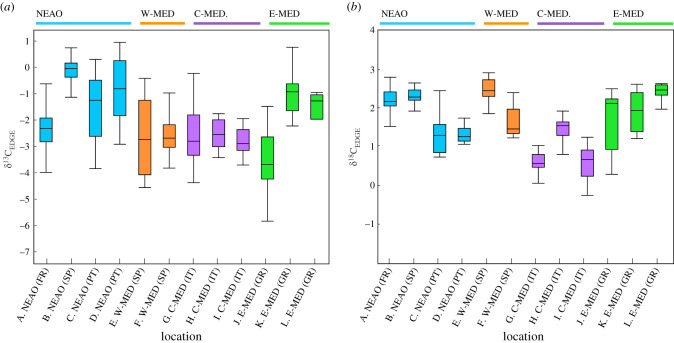
Box plot of cuttlebone (*a*) δ^13^C_EDGE_ and (*b*) δ^18^O_EDGE_ values by site and region for of *Sepia officinalis*. Different colour box plots shown for the four major regions.

Similarly, spatial variation in cuttlebone δ^13^C_EDGE_ and δ^18^O_EDGE_ values (recent life history) were detected for *S. officinalis* (MANOVA, *p* < 0.01; figure 4). Mean cuttlebone δ^13^C_EDGE_ values ranged from −3.49‰ to −0.02‰ across the 12 sites sampled. Again, a discernible NEAO versus Mediterranean Sea pattern was observed for cuttlebone δ^13^C_EDGE_, with mean values significantly higher in the NEAO (−0.73‰ ± 1.10‰) relative to the three regions within the Mediterranean Sea: W-MED (−2.70‰ ± 0.87‰), C-MED (−2.59‰ ± 0.80‰), E-MED (−2.21‰ ± 1.60‰) (ANOVA, *p* < 0.01, Tukey HSD *p* < 0.05). Spatial variation in cuttlebone δ^18^O_EDGE_ was also pronounced across the 12 sites (range: 0.50‰ to 2.42‰). Although a significant region effect was observed (ANOVA, *p* < 0.01), *S. officinalis* sampled from the NEAO and two regions within the Mediterranean Sea displayed similar cuttlebone δ^18^O_EDGE_ values: NEAO (1.88‰ ± 0.54‰), W-MED (1.64‰ ± 0.37‰) and E-MED (1.91‰ ± 0.63‰) (Tukey HSD *p* > 0.05). Region-specific variation in cuttlebone δ^18^O_EDGE_ was due to *S. officinalis* sampled from the C-MED having significantly lower δ^18^O_EDGE_ values (0.93‰ ± 0.55‰) relative to the other three regions (Tukey HSD *p* < 0.05).

Paired comparisons of cuttlebone core and edge material were also assessed to determine whether ontogenetic changes in habitat or environmental conditions may have occurred between early and recent life-history periods. Although general regional trends persisted for both cuttlebone core and edge samples, differences were detected between the two sections of the cuttlebone assayed for both markers. Cuttlebone δ^13^C_CORE_ and δ^13^C_EDGE_ values were significantly different (paired *t*-test, *p* < 0.01), and mean cuttlebone δ^13^C_EDGE_ values were approximately 2‰ higher than δ^13^C_CORE_ values (mean difference ± 1 s.d.: 2.03‰ ± 1.48‰). Observed differences were highly variable among *S. officinalis* from the 12 sampling sites (figures 3 and 4). Similarly, differences between the early and recent life-history periods were observed for δ^18^O_,_ and cuttlebone δ^18^O_EDGE_ values were significantly higher than δ^18^O_CORE_ values (mean difference 1.00‰ ± 0.93‰).

### 3.2. Classification to natal sites and stock discrimination

**Figure 5.**
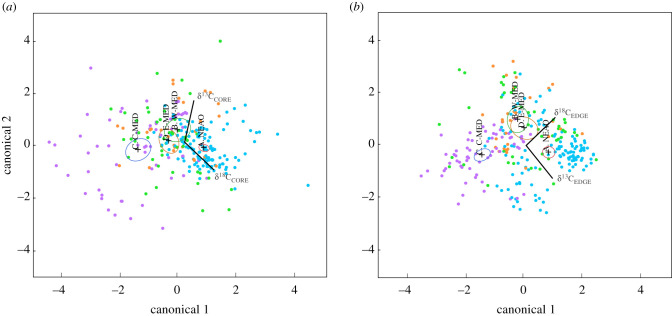
Canonical discriminant analysis based on cuttlebone δ^13^C and δ^18^O values of *Sepia officinalis* from the four regions: NEAO, W-MED, C-MED, E-MED. Ninety-five percent confidence ellipse around each centroid (denoted with + symbol) shown for cuttlebone (*a*) core and (*b*) edge. Biplot vectors from each centroid indicate the influence of δ^13^C and δ^18^O on regional discrimination.

Canonical discriminant analysis based on cuttlebone δ^13^C and δ^18^O of *S. officinalis* showed clear separation of 95% confidence ellipses for each centroid based on cuttlebone core (figure 5*a*) and edge (figure 5*b*) values. Biplot vectors from a grand mean indicated that discrimination to the four regions was influenced by both δ^13^C and δ^18^O. QDFA based on cuttlebone δ^13^C_CORE_ and δ^18^O_CORE_ (early life history) of *S. officinalis* resulted in cross-validated classification success of 62.1% to the four geographical regions. QDFA parameterized with δ^13^C_EDGE_ and δ^18^O_EDGE_ (recent life history) showed a slight reduction in classification success to 60.2%. Combining cuttlebone material from the two life-history periods (core and edge) resulted in the highest cross-validated classification success (75.4%) to the four regions, indicating that regional-specific chemical differences in cuttlebone δ^13^C and δ^18^O at both life-history periods were useful for discriminating *S. officinalis* from the geographical areas sampled. We also ran QDFA on a subset of sites in both the NEAO (site C, D) and W-MED (sites E, F) to evaluate the suitability of using δ^13^C_CORE_ and δ^18^O_CORE_ signatures for assessing population exchange, and classification success to these two regions was 84.5%.

### 3.3. Population connectivity between NEAO and Mediterranean Sea

**Figure 6.**
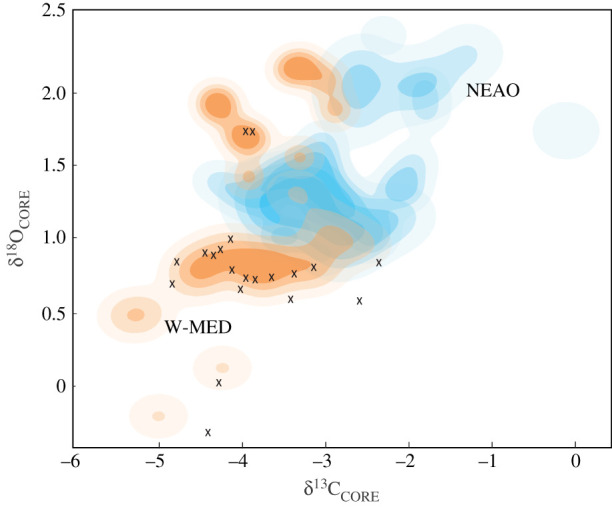
Contour plots of cuttlebone δ^13^C and δ^18^O values for *Sepia officinalis* for the two locations nearest to the Strait of Gibraltar in both the NEAO (sites C and D) and W-MED (sites E and F) was used for prediction of natal origin. Bivariate kernel density estimated at four levels for samples from both NEAO and W-MED (25%, 50%, 75% and 100%). Cuttlebone δ^13^C and δ^18^O values for *S. officinalis* of unknown origin collected near the Strait of Gibraltar are shown with X symbol.

Predictions of natal origin for *S. officinalis* collected in close proximity to the Strait of Gibraltar (Gulf of Cadiz) were used to assess potential exchange (i.e. movement) of individuals between the NEAO and Mediterranean Sea. A modified baseline comprised only of cuttlebone δ^13^C_CORE_ and δ^18^O_CORE_ values for individuals collected from two sites closest to the Strait of Gibraltar in both the NEAO and Mediterranean Sea (NEAO = C, D; W-MED = E, F) was used to predict the natal origin of *S. officinalis* from the Gulf of Cadiz. The majority of δ^13^C_CORE_ and δ^18^O_CORE_ values of *S. officinalis* collected in the Gulf of Cadiz matched bivariate kernel density estimates of cuttlebone δ^13^C_CORE_ and δ^18^O_CORE_ for individuals from the W-MED (E, F) rather than NEAO (C, D) (figure 6). MLE-based predictions of natal origin using the modified baseline indicated that *S. officinalis* in our Gulf of Cadiz sample were almost exclusively of western Mediterranean origin (95.4% ± 8.2%).

## 4. Discussion

Spatial variation in cuttlebone δ^13^C and δ^18^O of *S. officinalis* was observed across the large geographical scale investigated, with salient regional-specific differences in geochemical signatures present for both early (core) and recent (edge) life-history periods detected. Classification to the four regions (NEAO, W-MED, C-MED, E-MED) based on mixed-stock models parameterized with cuttlebone δ^13^C and δ^18^O was also similar using core or edge signatures. Regional discrimination based on these natural markers indicated that environmental conditions and drivers of seawater and/or cuttlebone geochemistry likely function at broad spatial scales in the NEAO and Mediterranean Sea. The four-region framework adopted for our study appears to adequately capture major geographical trends in cuttlebone geochemistry at spatial scales that are often isotopically distinct within marginal seas [20,21].

Noticeable trends in cuttlebone δ^13^C of *S. officinalis* were present among sites and regions, although site to site variability within most regions was relatively modest, particularly for δ^13^C_CORE_. A notable trend included cuttlebone δ^13^C_CORE_ values being more positive for individuals collected from nearly all sites in the NEAO (A-D) relative to sites within the Mediterranean Sea (E-L). We also observed a gradient of decreasing δ^13^C_CORE_ values from the NEAO to the C-MED, followed by a slight increase at sites in the E-MED. Similar to our findings, conspicuous differences in phytoplankton δ^13^C (δ^13^C_PHY_) and zooplankton δ^13^C (δ^13^C_ZOO_) have been reported between areas of the NEAO and Mediterranean Sea where *S. officinalis* were collected, with δ^13^C values more positive in the NEAO relative to the Mediterranean Sea [21]. This may be attributed to differences in primary productivity and phytoplankton growth rates that are reported to be higher in areas sampled in the NEAO, possibly leading to higher δ^13^C_PHY_ in this region [22]. In addition, it is possible that cuttlebone δ^13^C_CORE_ values may be influenced to some degree by maternally derived geochemical signatures, which can be conserved in the cores of biogenic hard parts [23]. We also observed generally higher δ^13^C_CORE_ and δ^13^C_EDGE_ values for *S. officinalis* collected in the E-MED relative to the C-MED and W-MED, and this appears due to higher water temperatures (increases phytoplankton growth rate) and elevated salinity in the E-MED; both parameters are positively related to δ^13^C_PHY_ [24]. Finally, it is important to note that sites within each region, particularly for δ^13^C_CORE_, generally maintained similar mean values, particularly in the NEAO, C-MED and E-MED. This is noteworthy because sites within each respective region were often separated by considerable distances (greater than 500 km) and in different marginal seas (e.g. Tyrrhenian and Adriatic in C-MED), suggesting that the resolution of this marker appears to be functional at the geographical scale investigated (i.e. four regions).

Regional distinctions in cuttlebone δ^18^O_CORE_ and δ^18^O_EDGE_ values between sites in the NEAO and Mediterranean Sea were also detected, and it is assumed that δ^18^O in biogenic carbonates reflects seawater (δ^18^O_SEAWATER_) values at the ocean-basin scale [25]. Patterns in cuttlebone δ^18^O for *S. officinalis* did not entirely match previously reported gradients in δ^18^O_SEAWATER_ for offshore waters in the NEAO and Mediterranean Sea [20,26], suggesting that other physico-chemical or biological factors likely play a role in determining cuttlebone δ^18^O. The mismatch between δ^18^O_SEAWATER_ and cuttlebone δ^18^O_CORE_ is possibly due in part to *S. officinalis* occupying inshore/near shore waters during early life [3], and such areas are often heavily influenced by freshwater inflow which alter δ^18^O values. Therefore, δ^18^O_SEAWATER_ values in near shore nursery habitats of *S. officinalis* may not align well with predictions from ocean models used to predict geographical variation in δ^18^O_SEAWATER_. The most noticeable discontinuity in cuttlebone δ^18^O_CORE_ occurred between sites in the NEAO and Mediterranean Sea. Also, both cuttlebone δ^18^O_CORE_ and δ^18^O_EDGE_ values for sites in the E-MED off Greece were generally higher compared to all other sites in the Mediterranean Sea, which is not unexpected because δ^18^O_SEAWATER_ is predicted to be highest in the eastern part of this marginal sea [20]. Elevated δ^18^O_SEAWATER_ and cuttlebone δ^18^O_CORE_ and δ^18^O_EDGE_ in the E-MED is assumed to be due to increased salinity (evaporation), which causes a disproportionate removal of the lighter isotope (^16^O) in water vapour, leaving behind more of the heavier isotope and resulting in higher overall δ^18^O_SEAWATER_ [27].

Classification success to the four regions sampled using cuttlebone δ^13^C and δ^18^O for early (core) and recent (edge) life-history periods indicated that *S. officinalis* collected from the NEAO, W-MED, C-MED and E-MED experienced different environmental conditions. We also observed that cuttlebone δ^13^C and δ^18^O values at sites within a region were often statistically similar, supporting the delineations used in our regional framework. This also indicates that environmental conditions experienced by *S. officinalis* were distinctive at the regional scale, and possibly associated with population structure. A recent study using genetic markers reported that populations of *S. officinalis* in the Mediterranean Sea are fragmented into several subpopulations [28], and the geographical areas of their genetic clusters were remarkably similar to our regional resolution using geochemical markers. Interestingly, all of our sampling sites in both the C-MED (G, H, I) and E-MED (J, K, L) were each associated with unique genetic clusters (i.e. subpopulations). Similarly, moving into the western part of the basin and into the NEAO, the population structure again from genetics was similar to results from cuttlebone isotope geochemistry. Genetic data indicated the presence of two genetic clusters in the NEAO with a break between our collection sites in the Bay of Biscay (A, B) and sites near the Strait of Gibraltar (C, D). Similarly, we detected a shift in both cuttlebone δ^18^O_CORE_ and δ^18^O_EDGE_ between these two geographical areas (A, B versus C, D) (figures 4*b* and 5*b*). Genetic data also showed that sites on both sides of the Strait of Gibraltar (NEAO = C, D; W-MED = E) were part of the same subpopulation cluster, indicating that gene flow occurs through this corridor [28].

Application of these geochemical markers was also used to investigate exchange of *S. officinalis* between the NEAO and Mediterranean Sea over a restricted geographical scope. Previous mark–recapture and electronic tagging studies suggest that based on observed rates of movement (km day^−1^) *S. officinalis* are easily capable of travelling several hundred kilometers [3,5]. Therefore, a modified baseline was developed using a subset of sites proximal to the Strait of Gibraltar in both the NEAO and W-MED to assess potential population connectivity. Mixed-stock predictions of natal origin showed substantial exchange with individuals of W-MED origin presumably moving through the Strait of Gibraltar and into the NEAO. Migrations from natal sites in the Mediterranean Sea to areas in the NEAO occur for highly migratory species such European eel *Anguilla anguilla* [29] and Atlantic bluefin tuna *Thunnus thynnus* [30]; however, connectivity between the NEAO and Mediterranean Sea for species assumed to display limited movements is ostensibly uncommon, and this type of egress activity has not been previously reported for S*. officinalis*. Nevertheless, our observation of *S. officinalis* of W-MED origin being present in the NEAO proximal to the Strait of Gibraltar is in accord with findings on stock structure using genetic markers, with Drábková *et al*. [28] reporting that *S. officinalis* from the NEAO and W-MED on each side of this passageway were grouped into the same subpopulation (genetic cluster). Because *S. officinalis* lays egg clusters that are typically attached to benthic plants or substrate and hatch fully developed [31], their dispersal potential relative to species with pelagic larvae is likely very limited. Consequently, gene flow through the Strait of Gibraltar requires active movement of individuals that is likely facilitated by strong surface currents flowing through this corridor [32] and offshore overwintering movements [3], which supports our finding of W-MED origin recruits west of this corridor in the NEAO. Still, it is important to note that our sample was limited in size and therefore may not accurately represent the stock composition of W-MED and NEAO recruits in the Bay of Cadiz.

Finally, higher cuttlebone δ^13^C and δ^18^O in edge relative to core material is indicative of the ontogenetic shifts that typically occurs for *S. officinalis*, with juveniles often inhabiting bays, estuaries, or near shore environments [31]. Physico-chemical conditions in these nurseries (e.g. lower salinity and higher temperature) are influenced by near shore processes (e.g. freshwater inflow), which appear responsible for individuals displaying distinct δ^13^C and δ^18^O signatures. Increases in cuttlebone δ^13^C_EDGE_ and δ^18^O_EDGE_ at most of the sites surveyed is in accord with the shift of larger/older individuals to deeper, cooler and more saline offshore waters [8]. Increases in δ^18^O in the biogenic structures of bony fishes [33,34] and sharks [35] have also been attributed to inshore–offshore transitions or shifts in salinity exposure due to the positive relationship between salinity and δ^18^O_SEAWATER_ [36] and the inverse relationship of δ^18^O with temperature [8], which supports the cuttlebone core to edge change observed here for *S. officinalis.* Although ontogenetic shifts in cuttlebone δ^13^C and δ^18^O appear to correspond to inshore–offshore migrations, a fair degree of natural variability was present between core and edge signatures both within and across sites. Our finding is not surprising and due in part to the fact that physico-chemical differences between inshore–offshore waters can vary widely across broad geographical ranges [37]. It is also important to note that variability in both markers was more pronounced for cuttlebone edge material. This was likely due in part to the variation in the collection month and size/age composition of the sample at each site (table 1) because edge material did not always represent the same life-history interval or season while core material reliably characterized the early life period (nursery habitat) of all *S. officinalis* in our sample.

## 5. Conclusion

Our findings support the use of cuttlebone geochemistry to gain further insight into past environmental conditions and population connectivity of *S. officinalis*. Geographical variation in cuttlebone δ^13^C and δ^18^O implies that physico-chemical conditions experienced by *S. officinalis* during both early life and later periods in life are different among the four regions investigated. Given that environmental conditions are known to influence local adaptations (i.e. adaptive response) and population genetics [38], region-specific differences in environmental conditions experienced by *S. officinalis* may serve as a selective force leading to population structure (sub-populations) in the NEAO and Mediterranean Sea. Region-specific variation in cuttlebone δ^13^C and δ^18^O also provides additional insight regarding the spatial resolution and suitability of these geochemical markers for future investigations of the population connectivity of *S. officinalis* as well as highly migratory taxa with home ranges that include areas within and outside the Mediterranean Sea (e.g. Atlantic bluefin tuna; Rooker & Secor [39]). Although regional-scale variation in cuttlebone δ^13^C and δ^18^O was evident, we also demonstrate that the approach shows promise for applications at smaller spatial scales (site-specific) and reveal for the first time that *S. officinalis* with putative Mediterranean Sea (W-MED) signatures were observed in the NEAO, suggesting that individuals of Mediterranean origin may actively move through the Strait of Gibraltar and enter the NEAO.

## Data accessibility

Data are available as part of the electronic supplementary material.
